# An Innovative Method to Identify Autoantigens Expressed on the Endothelial Cell Surface: Serological Identification System for Autoantigens Using a Retroviral Vector and Flow Cytometry (SARF)

**DOI:** 10.1155/2013/453058

**Published:** 2013-01-16

**Authors:** Tsuyoshi Shirai, Hiroshi Fujii, Masao Ono, Ryu Watanabe, Tomonori Ishii, Hideo Harigae

**Affiliations:** ^1^Department of Hematology and Rheumatology, Tohoku University Graduate School of Medicine, 1-1 Seiryo-cho, Aoba-ku, Sendai, Miyagi 980-8574, Japan; ^2^Department of Histopathology, Tohoku University Graduate School of Medicine, 1-1 Seiryo-cho, Aoba-ku, Sendai, Miyagi 980-8574, Japan

## Abstract

Autoantibodies against integral membrane proteins are usually pathogenic. Although anti-endothelial cell antibodies (AECAs) are considered to be critical, especially for vascular lesions in collagen diseases, most molecules identified as autoantigens for AECAs are localized within the cell and not expressed on the cell surface. For identification of autoantigens, proteomics and expression library analyses have been performed for many years with some success. To specifically target cell-surface molecules in identification of autoantigens, we constructed a serological identification system for autoantigens using a retroviral vector and flow cytometry (SARF). Here, we present an overview of recent research in AECAs and their target molecules and discuss the principle and the application of SARF. Using SARF, we successfully identified three different membrane proteins: fibronectin leucine-rich transmembrane protein 2 (FLRT2) from patients with systemic lupus erythematosus (SLE), intercellular adhesion molecule 1 (ICAM-1) from a patient with rheumatoid arthritis, and Pk (Gb3/CD77) from an SLE patient with hemolytic anemia, as targets for AECAs. SARF is useful for specific identification of autoantigens expressed on the cell surface, and identification of such interactions of the cell-surface autoantigens and pathogenic autoantibodies may enable the development of more specific intervention strategies in autoimmune diseases.

## 1. Introduction

Inappropriate humoral and cellular immune responses mediate the tissue damage in autoimmune diseases, and the outcome of an autoimmune disease is influenced mainly by the tissue distribution of target self antigens [1]. The pathogenesis of most autoimmune diseases is highly complex and involves multiple cellular and humoral pathways. One part of the humoral arm of the immune assault is caused by autoantibodies, and the mechanisms of autoimmune damage mediated by many autoantibodies have been studied [2]. Clinically, specific autoantibodies are critical for the diagnosis, classification, and monitoring of autoimmune diseases [2]. 

Autoantibodies cause damage through a number of mechanisms, including the formation of immune complexes, cytolysis or phagocytosis of target cells, and interference with cellular physiology [3]. The cellular localization of the target antigen is believed to play a critical role in the pathogenetic potential of autoantibodies [4]. Intracellular proteins are preferential targets of autoantibodies in autoimmune diseases, but many questions remain unanswered regarding how autoantibodies against intracellular proteins play pathogenic roles. In contrast, it is generally accepted that autoantibodies against integral membrane proteins are usually pathogenic [1]. Some autoantibodies have been clearly confirmed to be pathogenic in several autoimmune diseases, and a model for customized and specific therapeutic approaches against a highly pathogenic subset of autoantibodies using small molecules have been reported [5]. 

In 1971, Lindqvist and Osterland first described autoantibodies to vascular endothelium based on indirect immunofluorescence (IIF) experiments [6]. These autoantibodies were called anti-endothelial cell antibodies (AECAs) and were defined as autoantibodies targeting antigens present on the endothelial cell (EC) membrane [7]. As target antigens of AECAs are present on the ECs, which are always in contact with these circulating antibodies, AECAs have the potential to induce vascular lesions directly. Here, we present a review of AECAs and a novel method for identification of cell-surface autoantigens.

## 2. AECAs

### 2.1. AECAs and Disease

The presence of AECAs has been reported in patients with a wide variety of diseases, including collagen diseases (Table 1), inflammatory bowel disease, diabetes, thyroid diseases, thrombotic thrombocytopenic purpura, primary sclerosing cholangitis, interstitial lung disease, chronic obstructive lung disease, uveoretinitis, renal transplantation, Susac syndrome, masked hypertension, and atherosclerosis [8–23]. AECAs are correlated to disease activity in some collagen diseases, and are thought to be critical especially for vascular lesions in collagen diseases [23]. In addition, AECAs have been shown to be clinical signs of vasculitis in patients with systemic lupus erythematosus (SLE) and rheumatoid arthritis (RA) [24]. AECAs were also reported to play critical roles in several pathophysiological conditions, including pulmonary hypertension, digital ulcers, and gangrene [21, 22].

AECAs are detected even in healthy subjects [25, 26]. These natural autoantibodies interact with living ECs with lower affinity as compared to pathologic AECAs, and their antigens are highly conserved protein families. They contribute to modulate endothelial function with protective anti-inflammatory and anti-thrombotic functions [26].

### 2.2. Detection and Identification of AECAs

Methods for detection of AECAs have not been standardized, and a number of methods have been reported, including IIF, cell-based-enzyme linked immunosorbent assay (ELISA), flow cytometry, radioimmunoassay, western blotting (WB), and immunoprecipitation [22, 23]. As these each of methods have advantages and disadvantages, use of different technical approaches to obtain more robust data is recommended [7].

Human umbilical vein endothelial cells (HUVECs) are commonly used as a substrate, but antigen patterns of ECs differ among other ECs, passage numbers, and culture conditions [27]. It is also important whether ECs are fixed or not because fixation induces permeabilization of the EC membrane, and intracellular antigens become accessible to antibodies [22]. The results of AECA positivity were therefore not considered in the same light, and the prevalence of AECAs differed among studies (Table 1). Miura et al. recently reported a novel solubilized cell-surface protein capture ELISA for detection of AECAs [28], and further evaluation and standardization are needed. 

### 2.3. Pathogenicity of AECAs

An experimental animal model for pathogenicity of AECAs was reported by Damianovich et al. [29]. In their experiment, BALB/c mice were actively immunized with the purified AECAs from a patient with granulomatosis with polyangiitis. Three months after a booster injection with human AECAs, mice developed endogenous AECAs, and histological examination of lungs and kidneys revealed both lymphoid cell infiltration surrounding arterioles and venules.

AECAs have been shown to be correlated with disease activities, and have the potential to induce vascular lesions because their targets are expressed on ECs that are readily accessible to these circulating antibodies. AECAs are also considered to play roles in the development of pathological lesions by a number of methods as described below [22, 23, 30–32].

The first is the cytotoxicity of ECs through complement-dependent cytotoxicity (CDC) and antibody-dependent cell-mediated cytotoxicity (ADCC). CDC activity of AECAs was reported in patients with SLE, Takayasu arteritis, hemolytic-uremic syndrome, and Kawasaki disease [7, 24, 33–35]. Recently, we confirmed that fibronectin leucine-rich transmembrane protein 2 (FLRT2) is a novel target antigen of AECAs in SLE, which exerts direct cytotoxic effects through CDC [9].

The second is the induction of coagulation. AECAs may exhibit procoagulant effects by the production of tissue factor in SLE and the release of heparin sulfate in systemic sclerosis (SSc) [36, 37].

The third is the induction of apoptosis. AECAs may induce EC apoptosis through CD95 or cross-reaction with anti-phospholipid antibodies [38–40]. Dieudé et al. reported that heat-shock protein (Hsp60) bound to ECs and induced phosphatidylserine exposure and then apoptosis [41]. Margutti et al. identified antibodies to the C-terminus of Ral-binding protein 1 (RLIP76), and these autoantibodies induced oxidative stress-mediated EC apoptosis [42].

The fourth is the activation of ECs. AECAs were reported to induce the secretion of interleukin (IL)-1*β*, IL-6, IL-8, and monocyte chemotactic protein-1, (MCP-1), and the expression of adhesion molecules such as E-selectin, intercellular adhesion molecule 1 (ICAM-1), and vascular cell adhesion molecule 1 (VCAM-1) [8, 24, 31], which cause leukocyte recruitment and adhesion. 

Alard et al. reported that recognition of cell-surface adenosine triphosphate (ATP) synthase in the low pH microenvironment contributes to intracellular acidification of ECs, which may induce cell death and trigger inflammation [43]. 

As described above, there is a great deal of evidence that AECAs play pathogenic roles in collagen diseases. Identification of targets of AECAs is required because (a) antigen-specific detection systems are important for establishing diagnostic tools and standardization of AECAs measurement, (b) identification will enable thorough analysis of the pathogenicity of AECAs, and (c) AECA-autoantigen interactions may be good targets for specific therapeutic approaches against highly pathogenic autoantibodies.

## 3. Technologies for Identification of Autoantigens for AECAs

The prevalence of AECAs varies according to the type of ECs used for detection [44]. It was demonstrated that AECAs cross-react with human fibroblasts [45], and partial inhibition of AECA activity was documented by absorption of the AECA-containing sera with mononuclear cells [8]. It was also reported that a structure shared by platelets and ECs was recognized by a subset of AECAs [46]. These data suggested that the target antigens of AECAs may include not only EC-specific but also non-EC-specific molecules.

Target antigens of AECAs have been investigated intensively, but they are heterogeneous, and the following classification of target antigens was proposed: membrane component, ligand-receptor complex, and molecule adhering to the plasma membrane [8]. The EC autoantigens may be either constitutively expressed or translocated from intracellular compartment to membrane by cytokines, such as IL-1 and tumor necrosis factor *α* (TNF*α*), or physical effects [8, 47]. The reported autoantigens and their pathogenicities are summarized in Table 2 [7, 9, 22–24, 42, 43, 47–56].

Several molecules can bind to ECs and are called “planted antigens” for AECA presumably via charge-mediated mechanisms, a DNA-histone bridge, or a specific receptor. Myeloperoxidase, DNA, and *β*2-glycoprotein I (*β*2-GPI) are thought to adhere to ECs during incubation of ECs with sera from patients. Extracellular matrix components, such as vimentin, may also be target antigens for AECAs [57]. Proteinase 3 (PR3) could represent another potential cryptic target antigen [58]. PR3 has been maintained to migrate to the plasma membrane of ECs, following stimulation [8].

As methods for identification of target antigens of AECAs, immunoprecipitation and WB of glycoproteins from the EC membrane with AECA-positive sera have been used [8, 23]. Although numerous protein bands were reported as candidates for target antigens by this method, some of the bands were considered to be artifacts [8], and further identification of given bands was also sometimes difficult.

Alternative methods have been developed, such as proteomics analysis using two-dimensional electrophoresis followed by matrix-assisted laser desorption ionization time of flight mass spectrometry [8, 23] and expression libraries [8, 42, 56]. 

Proteomics analysis identified vimentin, Hsp60, voltage-dependent anion-selective channel 1 (VDAC-1), peroxiredoxin 2, and ATP synthase as targets for AECAs [41, 43, 48–50]. Expression libraries also identified tropomyosin, T-plastin, and RLIP76 [42, 56], and these technologies are therefore promising. The problem is that most of the molecules reported to date as targets for AECAs are intracellular proteins (Table 2) although AECAs must be directed against the cell surface. These two methods are not specific for detecting cell-surface molecules rather than intracellular molecules. In addition, extraction of some membrane proteins has been reported to be difficult in proteomics analysis, and this may make it difficult to identify such proteins as AECA targets [7]. 

**Figure 1:**
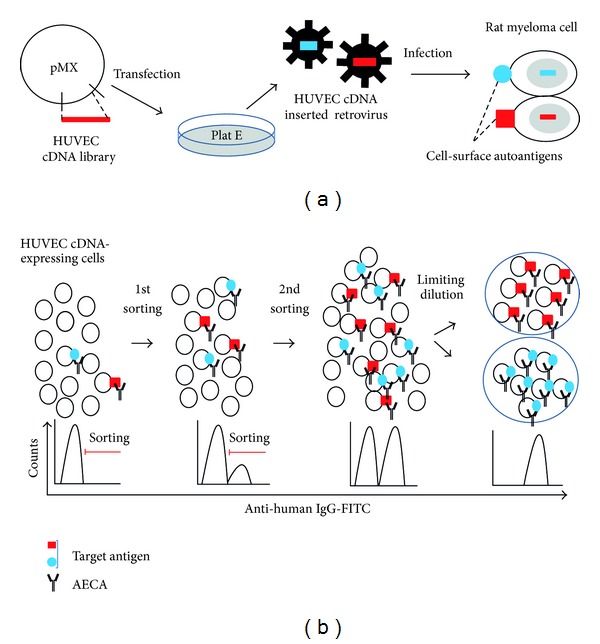
Serological identification system for autoantigens using a retroviral vector and flow cytometry (SARF). (a) Generation of human umbilical vein endothelial cell (HUVEC) cDNA-expressing cells. (b) Sorting of cells expressing cell-surface autoantigens.

To overcome this problem, we constructed a novel expression cloning system for specific identification of cell-surface antigens [9], which we call serological identification system for autoantigens using a retroviral vector and flow cytometry (SARF) (Figure 1), and we have confirmed that this system is useful to identify autoantigens expressed on the EC surface [9].

## 4. Strategy for Identification of Cell-Surface Autoantigens: SARF

### 4.1. Generation of HUVEC cDNA-Expressing Cells (Figure 1(a))

Our strategy to identify AECA target molecules involves use of a retroviral vector system and flow cytometry [9]. As described previously, antigen patterns of ECs differ among other ECs [27]. Because we used HUVECs as a substrate for AECAs measurement, we generated a HUVEC cDNA library using HUVECs grown in the same conditions as for AECAs measurement and ligated it into the retroviral vector, pMX [59]. Then, the HUVEC cDNA library in pMX was retrovirally transfected into the YB2/0 rat myeloma cell line [60]. As the localization of cellular molecules depends on their structures, only cell-surface molecules are expressed on the surface of YB2/0 cells transfected with the HUVEC cDNA library. 

### 4.2. Sorting of Cells Expressing Cell-Surface Autoantigens (Figure 1(b))

AECAs can bind only to cell-surface molecules in flow cytometry. Therefore, sorting of IgG-binding cells can concentrate and isolate cells expressing target molecules for AECAs on the cell surface. After staining of HUVEC cDNA-expressing YB2/0 cells with AECA IgG and secondary antibody, cells with strong fluorescent signals are sorted by flow cytometry. This step of sorting is repeated for several rounds to concentrate AECA IgG-binding cells. After concentration, several cell clones can be established from the AECA IgG-binding cell population by the limiting dilution method. 

### 4.3. Identification of Novel Cell-Surface Autoantigens

After polymerase chain reaction (PCR) amplification and cloning of HUVEC cDNA inserted into the genomic DNA of cloned cells, DNA sequencing can be performed followed by BLAST analysis, which enables the identification of the inserted cDNA. In this step, microarray analysis is an alternative method to identify the inserted cDNA. Next, an expression vector of the identified cDNA is generated and transfected into a cell line that does not express the identified protein. Finally, it is necessary to confirm that AECA IgG shows binding activity to 7-amino-actinomycin D-(7-AAD-) negative identified protein-expressing cells. If the binding activity is confirmed, it can be concluded that the identified protein is a novel autoantigen.

## 5. Novel Autoantigens Identified by SARF

### 5.1. FLRT2

We reported the membrane protein FLRT2 as a novel autoantigen of AECAs in patients with SLE based on results obtained using SARF [9]. FLRT2 is type I transmembrane protein located on the plasma membrane [61]. FLRT2 was shown to be expressed in the pancreas, skeletal muscle, brain, and heart with Northern blotting [61], and we confirmed the expression of FLRT2 on HUVECs and other ECs by flow cytometry and IIF [9]. Anti-FLRT2 antibody activity accounted for 21.4% of AECAs in SLE, and anti-FLRT2 activity was significantly correlated with low levels of complement C3, C4, and CH50 [9]. Anti-FLRT2 antibody induced CDC against FLRT2-expressing cells including ECs, indicating that anti-FLRT2 autoantibody may exhibit direct pathogenicity [9]. 

### 5.2. ICAM-1

**Figure 2:**
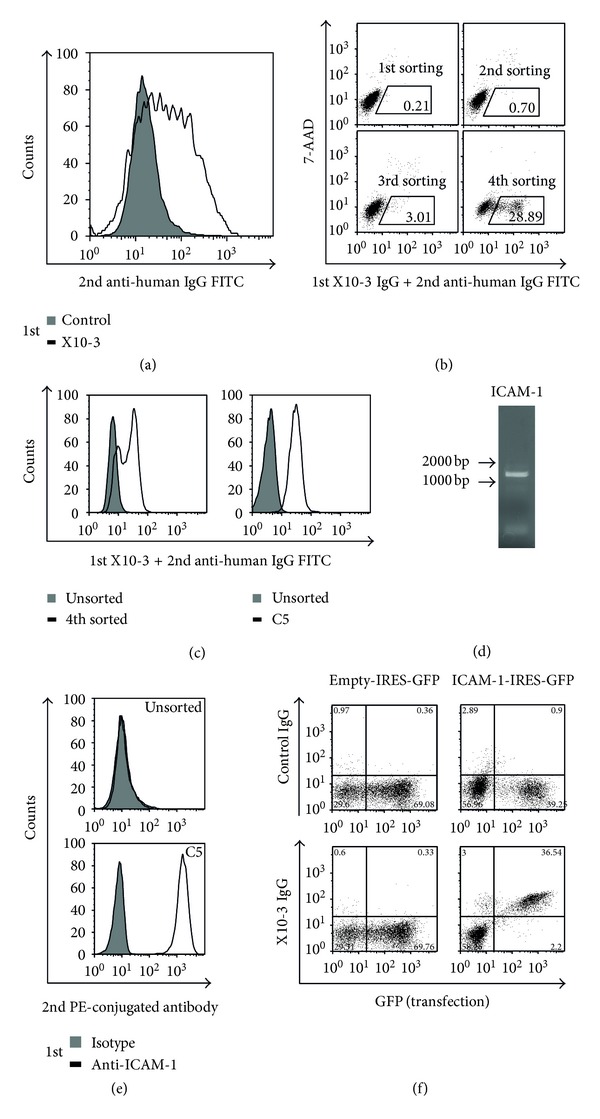
Identification of intercellular adhesion molecule 1 (ICAM-1) as a target antigen of anti-endothelial cell antibodies (AECAs). (a) Nonpermeabilized HUVECs were stained with 0.5 mg/mL of IgG of control or X10-3 from a patient with rheumatoid arthritis followed by secondary antibody and analyzed by flow cytometry. (b) HUVEC cDNA-expressing cells were stained with 0.5 mg/mL of X10-3 IgG followed by secondary antibody, and cells in the positive fraction were sorted (black box). (c) Unsorted and 4th sorted cells (left) and unsorted and cloned cells from 4th sorted cells, C5 (right), were stained with 0.5 mg/mL of X10-3 IgG followed by secondary antibody and analyzed by flow cytometry. (d) ICAM-1 cDNA fragments inserted into the genomic DNA of C5 were amplified, and PCR products were electrophoresed on an 0.8% agarose gel. (e) Unsorted and C5 were stained with isotype control or anti-ICAM-1 antibody, followed by secondary antibody and analyzed by flow cytometry. (f) Expression vector, empty-IRES-GFP, or ICAM-1-IRES-GFP were transfected into YB 2/0 cells, and these cells were stained with 0.5 mg/mL of control IgG or X10-3 IgG, followed by secondary antibody and analyzed by flow cytometry.

As AECAs can be detected in patients with collagen diseases, especially SLE, RA, and Takayasu arteritis [9], we further attempted to identify the autoantigens using SARF. One sample (X10-3) from an RA patient showed strong AECA activity (Figure 2(a)), and we selected this serum sample as the prototype of AECA for subsequent cell sorting. Using SARF, HUVEC cDNA-expressing YB2/0 cells were stained with X10-3 IgG and fluorescein isothiocyanate-(FITC-) conjugated secondary antibody, and cells with strong FITC signals were sorted by flow cytometry (Figure 2(b)). After the 4th sorting, cells bound to X10-3 IgG were markedly increased (Figure 2(c), left), and the C5 clone was established from the X10-3 IgG-binding cell population by the limiting dilution method (Figure 2(c), right). Microarray analysis revealed that the signal of ICAM-1 was significantly increased (2^6.16^-fold), and we confirmed that the ICAM-1 cDNA was inserted into the genomic DNA of X10-3-C5 clone (Figure 2(d)). We also confirmed the expression of ICAM-1 on the X10-3-C5 clone (Figure 2(e)). Next, we generated an expression vector for ICAM-1, which was transfected into YB2/0 cells. X10-3 IgG showed significant binding activity to 7-AAD-negative ICAM-1-expressing YB2/0 cells (Figure 2(f)), indicating that X10-3 IgG has anti-ICAM-1 activity. Thus, the membrane protein ICAM-1 was identified as a novel autoantigen of AECA in RA. ICAM-1 is an immunoglobulin-(Ig-) like cell adhesion molecule expressed by several cell types, including leukocytes and ECs. ICAM-1 plays an important role in both innate and adaptive immune responses. It is involved in the transendothelial migration of leukocytes to sites of inflammation, as well as in interactions between antigen presenting cells (APC) and T cells (immunological synapse formation) [62].

ICAM-1 was also confirmed to transduce signals “outside in” [63, 64]. The cross-linking of ICAM-1 with monoclonal antibodies was reported to activate the mitogen-activated protein kinase (MAPK) kinases ERK-1/2 and/or JNK [65–67]. The activation of ERK-1 lead to AP-1 activation [66], the ERK-dependent production and secretion of IL-8 and RANTES [67], and upregulation of VCAM-1 on the cell surface [66, 68]. ICAM-1 cross-linking can also upregulate tissue factor production [69] and proinflammatory cytokines, including IL-1 [70]. Lawson et al. reported production of anti-ICAM-1 IgM after cardiac transplantation, and the antibody induced robust activation of the ERK-2 MAPK pathway [71]. The use of anti-ICAM-1 antibody was examined for the treatment of RA, but the second course of therapy was associated with adverse effects suggestive of immune complex formation [72]. Identification of anti-ICAM-1 antibody in a patient with RA suggested that this autoantibody may exhibit such pathogenic roles. 

### 5.3. Pk (Gb3/CD77)

Using serum from an SLE patient who showed hemolytic anemia, SARF revealed that cDNA inserted into the cloned cells that were sorted with this AECA-IgG was alpha 1,4-galactosyltransferase (A4GALT). This AECA showed significant binding activity to 7-AAD-negative A4GALT-overexpressing YB2/0 cells. The A4GALT locus encodes a glycosyltransferase that synthesizes the terminal Gal*α*1-4Gal of Pk (Gb3/CD77) glycosphingolipid [73, 74]. This means that synthesis of the terminal Gal*α*1-4Gal is needed for the binding of this AECA-IgG. 

Gb3 is the Pk blood group antigen and has been designated CD77 [74]. Monoclonal antibodies against Pk (Gb3/CD77) are used as markers for Burkitt's B-cell lymphoma and are able to initiate apoptosis [75]. Pk (Gb3/CD77) plays a direct role in the entry of Shiga toxin into the cell [76], and the presence of Pk (Gb3/CD77) in the ECs of the kidney accounts for the development of hemolytic uremic syndrome during bacterial infection with Shigella species that produce verotoxin [77]. The anti-Pk (Gb3/CD77) antibody was reported to cause acute intravascular hemolytic transfusion reactions and recurrent spontaneous abortions due to damage to the placenta [73, 78]. These data suggested that Pk (Gb3/CD77) is one of the target antigens of AECAs in SLE patients manifesting hemolytic anemia, and that anti-Pk (Gb3/CD77) antibody may exhibit some pathogenic roles. 

Identification of A4GALT indicated the usefulness of SARF, which can be used to identify genes that encode not only the membrane protein itself, but also the transferase(s) responsible for modifying the membrane protein. 

As described above, this system is very useful for identification of cell-surface autoantigens. Although this system seems to present difficulties in sorting cells at very low frequency, we could isolate and clone autoantigen-expressing cells by repeated sorting.

As AECAs are a heterogeneous group of autoantibodies that target ECs, it is predicted that there are different autoantigens. Thus, it is important to determine the clinical significance and potential pathogenicity of identified autoantibodies. If an autoantibody is specific for a disease or pathophysiology, it could be used as a marker for diagnosis or classification according to the underlying pathophysiology. At the same time, the pathogenic potential of the autoantibody should also be examined. Along with in vitro studies mentioned previously, experimental animal models of identified autoantibody should be constructed to determine the pathogenetic reactions in vivo.

## 6. Summary

AECAs are considered to be critical, especially for vascular lesions in collagen diseases, but most are directed against molecules localized within the cell and not expressed on the cell surface. In addition to conventional immunoprecipitation and WB, proteomics and expression library analyses have been performed to identify the targets for AECAs with some success. SARF was developed to identify autoantigens expressed on the EC surface with greater sensitivity. Using SARF, we successfully identified three different membrane proteins as targets for AECAs: FLRT2 from patients with SLE, ICAM-1 from a patient with RA, and Pk (Gb3/CD77) from an SLE patient with hemolytic anemia. Using this technology, it may be possible to determine cell-surface autoantigens of AECAs and achieve a comprehensive understanding of AECA-mediated vascular injury. Furthermore, SARF can be used when autoantibodies against cell-surface molecules are considered to take part in autoimmune diseases. The identification of such pathogenic autoantibodies may enable the development of more specific intervention strategies in autoimmune diseases.

## Figures and Tables

**Table 1:** Prevalence of anti-endothelial cell antibodies.

Disease	% of positive sera
Systemic lupus erythematosus	15–85
Rheumatoid arthritis	0–87
Mixed connective tissue disease	33–45
Systemic sclerosis	15–84
Polymyositis/dermatomyositis	44–64
Antiphospholipid syndrome	0–64
Sjögren's syndrome	24-25
Polyarteritis nodosa	50–56
Microscopic polyangiitis	2–60
Granulomatosis with polyangiitis	19–80
Eosinophilic granulomatosis with polyangiitis	50–69
Takayasu arteritis	54–95
Giant-cell arteritis	33–50
Behçet's disease	14–80
Kawasaki disease	65

**Table 2:** Reported target antigens of anti-endothelial cell antibodies.

Disease	Target antigen	Pathogenicity
Systemic lupus erythematosus	DNA-DNA-histone	
	Ribosomal P protein PO	
	Ribosomal protein L6	
	Elongation factor 1-alpha	
	Adenylyl cyclase-associated protein	
	Profilin 2	
	Plasminogen activator inhibitor	
	Fibronectin	
	Heparan sulfate	
	*β*2-glycoprotein I	
	Heat-shock protein 60 (Hsp 60)	Apoptosis
	Heat-shock protein 70 (Hsp 70)	
	Fibronectin leucine-rich transmembrane protein 2 (FLRT2)	Complement-dependent cytotoxicity

Mixed connective tissue disease	Voltage-dependent anion-selective channel 1 (VDAC-1)	

Systemic sclerosis	Topoisomerase I	
	Centromere protein B (CENP-B)	

Vasculitis	Proteinase 3	
	Myeloperoxidase	
	Peroxiredoxin 2	Cytokine secretion
	Adenosine triphosphate (ATP) synthase	Intracellular acidification

Microscopic polyangiitis	Human lysosomal-associated membrane protein 2	

Behçet's disease	Alpha-enolase	
	C-terminus of Ral-binding protein 1 (RLIP76)	Apoptosis

Kawasaki disease	Tropomyosin	
	T-plastin	

Transplantation	Vimentin	
	Keratin-like protein	

Thrombotic thrombocytopenic purpura	Glycoprotein CD36	

Heparin-induced thrombocytopenia	Platelet factor 4 (PF4)	
	Heparin sulfate	
